# Failure to flow: An exploration of learning and teaching in busy, multi-patient environments using an interpretive description method

**DOI:** 10.1007/s40037-017-0384-7

**Published:** 2017-11-08

**Authors:** Teresa M. Chan, Kenneth Van Dewark, Jonathan Sherbino, Alan Schwartz, Geoff Norman, Matthew Lineberry

**Affiliations:** 1 Division of Emergency Medicine, Department of Medicine, McMaster University, Hamilton, Ontario Canada; 2 Department of Emergency Medicine, University of British Columbia, Vancouver, Ontario Canada; 3 Department of Medical Education, University of Illinois, Chicago, USA; 4 Department of Clinical Epidemiology & Biostatistics, McMaster University, Hamilton, Ontario Canada; 5 Department of Health Policy & Management, University of Kansas Medical Center, Kansas City, KS USA

**Keywords:** Qualitative research, Multi-patient environments, Clinical teaching

## Abstract

**Introduction:**

As patient volumes continue to increase, more attention must be paid to skills that foster efficiency without sacrificing patient safety. The emergency department is a fertile ground for examining leadership and management skills, especially those that concern prioritization in multi-patient environments. We sought to understand the needs of emergency physicians (EPs) and emergency medicine junior trainees with regards to teaching and learning about how best to handle busy, multi-patient environments.

**Method:**

A cognitive task analysis was undertaken, using a qualitative approach to elicit knowledge of EPs and residents about handling busy emergency department situations. Ten experienced EPs and 10 junior emergency medicine residents were interviewed about their experiences in busy emergency departments. Transcripts of the interviews were analyzed inductively and iteratively by two independent coders using an interpretive description technique.

**Results:**

EP teachers and junior residents differed in their perceptions of what makes an emergency department busy. Moreover, they focused on different aspects of patient care that contributed to their busyness: EP teachers tended to focus on volume of patients, junior residents tended to focus on the complexity of certain cases. The most important barrier to effective teaching and learning of managerial skills was thought to be the lack of faculty development in this skill set.

**Conclusions:**

This study presents qualitative data that helps us elucidate how patient volumes affect our learning environments, and how clinical teachers and residents operate within these environments.

## What this paper adds

This paper provides insights into how emergency physicians conceptualize busy, multi-patient environments. It describes the present ‘status quo’ of how one group of academic emergency physicians and residents define, deal with, and create learning experiences in multi-patient environments. This paper highlights areas for discussion that have not been previously discussed—namely how our postgraduate trainees and teachers are being affected by higher patient volumes and increasing complexity within our emergency diagnosis systems.

## Introduction

### Background

Patient volumes are increasing in health systems around the world. The concept of patients ‘flowing’ efficiently through the emergency department is fast becoming a critical skill for emergency physicians (EPs). Emergency department crowding is steadily increasing in the Western world and its negative impact on patient care is well documented [1–5]. Surveying the literature, one can find a burgeoning body of work that focuses on EP decision-making in the context of busy environments [6–11]. This literature brings in perspectives from other fields; for instance, engineers have used a human factors lens, examining and detailing workflow processes [6, 8], other papers adopt a business management approach [10] and other studies take an epidemiological approach to the problem [9]. However, few studies have specifically examined the impact of contextual pressures such as increased emergency department crowding on clinical teaching and learning, and how education unfolds in these environments.

### Ramifications for training

Critical to success as an EP is the ability to manage multiple competing demands simultaneously [12]. Trainees in programs such as emergency medicine are required to develop strong clinical skills treating a wide spectrum of disease while also learning to resuscitate patients of all ages. Additionally, they must implement emergency department management skills such as task prioritization, time management, coordination of teams, and negotiation skills to ensure safe and effective patient flow. Hence, emergency medicine training programs must teach important managerial and leadership skills to ensure trainees are successful, competent physicians when they begin unsupervised practice.

The ramifications of increased patient volumes and emergency department crowding for emergency medicine training are not well studied, but have the potential to be significant [13]. While there have been some studies on what emergency medicine teachers might teach [14] or how they balance teaching and clinical care [15, 16], the emergency department presents a microcosm within academic medicine that might shed light on how other specialties might similarly handle increased patient-care demands. Exploring how variable systemic and contextual pressures affect EPs’ and residents’ clinical teaching and learning environments may help us to better understand how trainees might learn despite busyness, but also how they learn to manage its impacts to mitigate the effects of such pressures on patients [13].

Our present study has two main goals: 1) To better understand how EPs and residents experience busy, crowded emergency department environments, and 2) To better grasp how these multi-patient situations affect the EP teachers’ and residents’ teaching and learning experiences.

## Method

### Study design

This study was part of a larger program of research exploring physician cognition in multi-patient environments using a meta-framework known as cognitive task analysis [17]. Cognitive task analysis incorporates multiple analytic techniques to create a holistic understanding of cognition around a phenomenon. The approach allows investigators to pragmatically apply several methods to create a construct of what participants think when they experience a phenomenon or encounter a certain problem. A cognitive task analysis incorporates three phases: knowledge elicitation, analysis, and knowledge representation.

This study is an analysis of our knowledge elicitation phase of the cognitive task analysis [17]. In general, the knowledge elicitation phase aims to help investigators understand how participants think about a given topic. For this aspect of our program of research, we conducted a qualitative study, using a semi-structured interview with an interpretive description technique for analysis. This enabled us to understand the recalled experiences of the teachers and trainees we interviewed. The interpretive description technique was utilized because our analytic team involved both a trainee and a teacher, and this technique has most often been applied in settings where practitioners are examining their own environments [18].

### Study setting and population

This study was conducted at the three teaching hospitals affiliated with McMaster University in Hamilton, Ontario, Canada. The annual emergency department visit rates for these three sites range from 40,000 to 65,000 visits per year, with an approximate admission rate of 20% and a hospital occupancy between 95–105%. We recruited a purposive sample of 10 peer-nominated EPs via a peer referral sampling technique [19] as well as a convenience sample of 10 junior EM trainees (i. e. postgraduate year 1 or 2) from our institution’s residency program. We intentionally sampled to ensure we had a wide breadth of experience in these environments by sampling from a pool of junior residents and practising, respected EP teachers. Peer sampling was used to ensure that EP teachers who were perceived by their peers as being respected for their efficiency were incorporated in this study.

### Study protocol

The interview guide (Table 1) was adapted from the work of Wiles [20] and modified using Klein’s *Eight Dimensions of Expertise* [17] to create further prompts for participants if they were not forthcoming with illustrative or elaborative answers as needed. The interview questions were then piloted with members of the investigatory team. Interviews were conducted from January 2014 to April 2015 by a single, experienced interviewer (TC). Interviews were between 25–45 minutes in duration. All participants provided informed consent. A contracted professional medical research transcriptionist transcribed each interview, removing any identifiers. During the initial part of the interview, participants were asked to describe challenging emergency department situations (see Table 1 for details).

**Table 1:** Interview guide

**Question 1:** Think back to a recent busy emergency department situation that was challenging for you—one in which you were asked to make difficult clinical decisions about how to proceed next with your overall management of the emergency department. Please share that experience with me
Prompt 1: Tell me about what was concerning or challenging to you. a. What was your gut telling you? b. What did you notice about the scenario that made it stand out?
Prompt 2: Tell me how you proceeded with emergency department management that day? a. What are the big picture issues that you take into consideration? b. What cues did you use to help fine-tune your approach?
Prompt 3: How did you evaluate the outcomes of the plan that you chose?
Prompt 4: As you think back what do you notice differently now? What stands out to you?
Prompt 5: What, if any, equipment do you use to help you plan your actions in the emergency department?
**Question 2:** When you are working with other doctors that are less experienced than you such as residents, how do you incorporate them into your management strategies?

### Ethics

Our study was granted approval by our institutional review board (Hamilton Integrated Research Ethics Board, #11-409).

### Data management and analysis

Basic demographics were collected from participating individuals. The transcripts of the interviews were analyzed using an inductive technique akin to the interpretive description technique [18]. The transcripts were iteratively reviewed by two investigators (TC, KVD), with concepts and memos constructed independently and then discussed periodically. These two members of the team were selected since one was a practising EP teacher and the other was a resident, allowing them to view the data in an interpretive manner. These concepts and memos were coded to create themes, which were merged and recombined as needed to best represent the data. Disagreements were resolved by a consensus building process, involving one other non-physician member of the investigatory team where appropriate to ensure reflexivity of the analysis team (ML). We actively sought out quotes that deviated from our coded themes with each successive session of analysis, and incorporated these divergent codes into amended versions of our codes by discussion and consensus building. We finalized the list of codes and themes once sufficiency was achieved, as determined by at least two transcripts yielding no additional codes or themes. Our analytic processes were documented in an audit trail, and the rest of the investigatory team were able to review these documents. To complement our analysis, we conducted a member check by sending our final analysis to our participants to ensure that the themes resonated and captured their ideas. No new themes were suggested during the member check. All attempts were made to adhere to the standards for reporting qualitative research [21].

## Results

### Demographics

The EP teachers and residents differed significantly in age, years of practice, and proportion of time spent in an academic centre, because some teachers had community-based practice. See Table 2 for details.

**Table 2:** Participant demographics

	Attending emergency physician teachers	Residents
Average age (years)	38.7 ± 5.4	29.0 ± 2.4
Average years in practice	12.0 ± 4.7	1.5 ± 0.5
% time spent at an academic centre	96.0 ± 12.6%	99.5 ± 1.6%

Means ± standard deviations are shown.

### Definition of challenging: ‘busyness’

There were four types of situations that the participants described in their descriptions of a busy, challenging day: 1) Single high-acuity cases, 2) High patient-volume days, 3) Multiple high-acuity cases being managed simultaneously, and 4) High-acuity case(s) in the setting of high patient-volume days with saturated resources. These themes related to experiences that our participants described readily as ‘busy’.

Interestingly, the residents tended to describe challenging and busy days through recollection of single-high-acuity cases or high patient-volume days. Unlike their more senior colleagues, the residents seldom considered the context of the ‘busy days’. Resident 1 stated:

*… [I]t was a busy night and there was a patient who came in with like (sic.) a decreased [level of consciousness] secondary to like (sic.) a hypoglycaemia and the question was whether or not to give the usual like (sic.) [50% Dextrose solution]. [H]owever this was a patient who had, like, a genetic condition which put them in, like, a very small weight, very small structure so it was like a forced decision and there was a lot of pressure from nursing… it was challenging and I debriefed with my staff and she was, like, I understand why in the moment.*

For example, EP teachers tended to focus more upon the multiplicity of tasks or systems-level issues, such as: nursing priorities, staffing issues (e. g., number of absentee staff), physical plant issues (e. g., location of sick patients), ‘bed-blocking’ (e. g. when admitted patients are held in the emergency department, and block a bed from being used by a new patient, also known as admission volume), and number of hand offs or handovers. Only one resident mentioned the waiting room or admission volumes, as opposed to the majority of EP teachers who noted these phenomena. This one resident (Resident 2) described a situation where multiple tasks were required of them:

*I was trying to balance a relatively busy department and make sure that people were seen and that disposition happened and that people were moving through. … It began to get busy once you had seen a few people and had to then keep track of their labs and their x‑rays and what was coming back … and making sure all of the consultants were phoning you back and actually seeing the patients who you had sent to them. So that was definitely very busy and I was task saturated.*

Another physician (Attending EP 3) vividly described multiple high-acuity scenarios in the context of a busy department:

*…I was working in a busy community emergency department in which there were 8 to 10 people waiting to be seen in the acute side …. and most of those patients were undifferentiated chest pain, abdominal pain or dyspnoea in the elderly. None of them were thought to be acutely over sick requiring acute resuscitation. [Three Emergency Medical Service crews subsequently] arrived within probably 6 to 7 minutes with one [vital signs absent] patient, a patient in rapid atrial fibrillation and a boarded and collared patient with moderate velocity [multiple vehicle collision]. The challenge was: 1) prioritizing the management of the patients; and, 2) managing flow in the emergency department…*

Another EP teacher (Attending EP 4) described how staffing and a highly emotional case affected the ability of the system to work well after a paediatric cardiac arrest case:

*[A] recent situation that was challenging for me …was … a very busy department, one other attending staff was there finishing charts, then it was myself, multiple handovers, lots of bed no admits (sic.) and we had a paediatric arrest come in. So the paediatric arrest was managed with the assist of the second doc that took a lot of resources and a lot of staff kind of emotion which definitely affected the flow of the department and it was a challenge in that it was something that was hard to pull back on training for necessarily.*

Generally, the attending EPs tended to describe more complex situations in their stories about challenging situations. These recollections incorporate systems-level thinking beyond the particulars of any individual case. Although they think about the individual cases, they tended to recall situations wherein they had to navigate through multiple cases in a situated fashion to attend to all the needs of the patients under their care. To achieve this aim the EP was harnessing all resources (nurses, learners) in order to do so. This is best exemplified by the following quote by Attending EP 2:

*I had some multiple septic patients who presented around the same time. I was the only staff physician at the time. I did have learners with me. So the way I dealt with it was I quickly eyeballed all of them, decided who was the sickest of them, and started assessing that patient, while at the same time ordering lab work and investigations and even broad-spectrum antibiotics [on the other patients]… The second sickest patient I sent my resident to see, and as we were the only people there the third and fourth patients could not be assessed however I had already eyeballed them and ordered investigations and the nurses were comfortable with that plan and they knew where to come and find me if any of the less sick patients became sicker. In that way we managed to assess, start resuscitation and manage all of them.*

### Factors that contribute to a ‘busy emergency department’

There were multiple factors that contributed to perceived busyness. These factors were found to be along three themes: 1) External factors (such as hospital resources), 2) Physician-related factors (attention, diligence, number of decisions needing to be made, reassessments, charting), and 3) Patient-related factors (single time-consuming cases, volume of patients, conflicting needs of different patients).

Residents tended to mention reassessments as a source of increased work, and therefore the perception of being busy. Specifically, they expressed concern over the unanticipated nature of these reassessments, and how the task of reassessment would often detract from their other patient care duties. They also more frequently mentioned interruptions or their difficulties with integrating information from multiple sources and using these to make decisions (i. e. being indecisive about having multiple ways in which to proceed). Unique to the residents’ interviews were situations where supervising physicians were sources of the ‘challenging’ situations. One resident described a situation where a supervising physician provided him with several patient charts all at once and asked him to prioritize their patient-care tasks: ‘*I was handed six charts and told how to prioritize them, to best see my patients. We also had 15 patients waiting in the waiting room that had to come in*.’ (Resident 4).

### Barriers to teaching prioritization and emergency department management skills

There were three themes that arose in the interviews regarding barriers to teaching and learning the tasks required to manage a busy emergency department. These barriers fell mainly into the following thematic areas: 1) Lack of teaching capacity in faculty, 2) Lack of foundational skills in the residents, and 3) Other competing interests.

The dearth of faculty development around facilitating the development of emergency department management and prioritization skills is of particular interest. Notably, faculty members were unable to name any specific formal faculty development to improve teaching around this skill set. They reflected that any teaching strategies they used were adopted from experiences as trainees themselves, or were techniques they derived via trial and error. The resident interviews revealed a similar paucity of formal teaching, noting that there was only some very cursory formal teaching around the Canadian Triage Acuity Score.

Some of the key barriers discussed were around the preparedness (or lack thereof) of junior residents to take on the responsibility of managing multiple patients. The attending EPs felt that the key barrier to learning about emergency department management was the lack of precursor skills such as handling individual cases properly. Lack of clinical exposure led to the failure of junior residents to identify sick patients, anticipate the needs of typical patients, and plan ahead. The following attending (Attending EP 7) best exemplifies this dilemma:

*There are times where if I’m working with PGY5 that I treat them like another staff person with some degree of supervision. And so in that circumstance, we might strategize about finding locales to work out of. But the vast majority of residents are not able to cope with a strategy like that and so they continue to see one patient at a time.*

Attending EPs also noted that junior residents were rarely exposed to basic tenets of task prioritization, and as such were unable to cope with multiple patients at once. Attending EPs recalled assuming more managerial roles to ‘deploy’ junior learners to accomplish tasks (i. e. managing one particular patient). Consequently, this may provide the trainee with fewer opportunities to assume a leadership role in the emergency department. Attending EPs often noted that junior learners tended to value individual cases over systems-level considerations. One EP teacher (Attending EP 4) noted a common scenario:

*[F]or example if there is a critical patient, I think that there is a tendency of junior learners to stay at the bedside forever and ever and ever with that patient… you have to remember that once you have stabilized the patient …you don’t have to remain at the bedside, that you need to get back and think about what else is going on in the department.*

Additionally, to be entrusted to manage the emergency department, EP teachers identified that residents needed to first understand and diagnose system-level problems. One EP (Attending EP 6) explained how he discusses situations with learners:

*… Sometimes it becomes a bit of a discussion point. So if we hear a patch about a patient that’s coming in, I will ask them, ‘Okay, there is a patient coming in. At the moment we have no resusc[itation] capability. We have no beds available. What would your strategy be to help open a bed?’*

If a learner did not display these precursor skills, EP teachers were unlikely to trust them to assume a more global managerial role. Relevant to this theme, many junior residents noted that they had minimal exposure to the basic tenets of task prioritization; they were often assigned duties by their EP teachers and were not prompted to take on multiple tasks at once. One EP explained (Attending EP6) how he assigned junior residents to single patients with higher perceived educational value:

*I will try and send them to see things that will have both educational value or may be interesting or may be relevant in terms of a procedure that a junior learner is looking to do. So, I will look at the tracker, figure out who it is I think has the most educational benefit… and I will send them to go and see those patients while I will go and see the patients who have less educational merit.*

There was a fairly prevalent theme around the presence of competing interests too. Attending EPs were apt to describe situations where there were tensions between efficiency and learning. For instance, residents were consistently described as being less efficient than attendings (Attending EP 9 described it best: *‘Likewise, if I’m working with very junior resident students, residents that I don’t know, often at that time, they become more of a slowdown…*’) Some attendings favoured taking on patients that they could see more efficiently, for example as Attending EP 8 states: ‘*Sometimes for psych[iatric] patients, they take a whole longer with them than I might, and so those patients it’s a whole lot easier if I just go see them myself.*’

## Discussion

EPs are regularly asked to manage multiple patients concurrently, and often must make time-sensitive decisions around the priorities across multiple patients. Our findings suggest that there are some key differences in perceptions of this particular skill set, which may inform some future work in this area.

### Differences between perceptions of EP teachers and residents

We discerned a difference in the way that junior physicians (residents) and EP teachers perceived challenging scenarios. The residents in our study tended to view single high-acuity scenarios as challenging cases; by contrast, EP teachers considered the complexities of the entire department within the greater healthcare system and how individual cases were situated within the complex emergency department scenario.

Gary Klein and his colleagues described two main macrocognitive phenomena that occur when experienced practitioners engage in their work in naturalistic environments: functions and processes [22, 23]. Bearing Klein’s work in mind, Schubert and colleagues found similar differences between the macrocognitive functions and processes of supervising EPs and their junior trainees [7]. From this work we know that expert EPs tend to be more intimately aware of how the emergency department fits into the larger health system [7]. EPs are also more focused on through-put, supervising learners, and applying their medical knowledge [7]. Novices tend to be more focused on specific patient care activities (e. g. charting, patient interview, workup, consultation/discharge processes), and not on the over-arching processes that EPs consider [7]. This may be purely a function of their situational awareness, but may also be an artifact of the difference in the roles played by these players within the system. In our study, we similarly found that attending physicians tend to focus on ‘running the emergency department’, and junior residents tend to be entrusted with caring for their small portfolio of patients. It appears that EP teachers tended to feel that it was important to allow junior residents to develop illness scripts [24] and learn to manage individual patients. Our participant answers reflected that EP teachers tend to restrict junior residents to managing their own smaller portfolio of patients, which explains the findings from Schubert’s study but also why our junior residents focused on challenges and busyness created by single-patient clinical scenarios. In contrast, EP teachers tended to view the single high-acuity cases in the context of the entire emergency department. These differences will need to be accounted for when designing educational interventions for teaching emergency department management skills, but also in planning transitional skills for those residents who are ready to move beyond managing individual patients. For instance, transition points in training may benefit from dedicated curricular planning. It may be useful to provide incoming senior residents with problem-based learning seminars, simulations, or workshops that ask them to prioritize between multiple patients and organize care for optimal efficiency. Since their duties as a junior are distinct from those expected of them as a senior (i. e. they are no longer just expected to manage their own portfolio of patients), spending time discussing multi-patient scenarios and how best to handle them may be useful for transitional trainees. Similarly, it may also be useful to reframe the focus of junior residency rotations or simulation curricula to generate rich experiences with common cases, to help establish the building blocks required for the residents to later flourish as senior residents.

### A case for staged progression towards multi-patient care

Of the barriers identified in our needs assessment, the most interesting finding was the perception by EP teachers that multi-patient care was necessarily preceded by the ability to provide good quality care for individual patients. Aligned with concepts of staged milestones [25] or entrustable professional activities [26], this suggests that EPs perceived there is a staged progression that requires a trainee to develop numerous precursor skills and accrue enough experience managing a single patient before they are entrusted with the care of multiple patients simultaneously or management of the entire emergency department.

### Need for more study and subsequent developing faculty

It was interesting to find that most of the participants were of the impression that prioritization was not a skill that was currently formally taught, but instead that it is a skill developed and learned in the clinical environment. By studying how faculty members have adapted to teach managerial skills, we can discover more robust strategies for teaching these skills, and translate this via faculty development to a broader range of teachers. Also, while experiential learning is important, there are possibly many avenues that may allow for teaching and development of this skill outside of the emergency department (e. g. teaching the Canadian Triage Acuity Score to junior learners in didactic sessions). Simulation, which has been used to teach about disaster management [27–30], or serious games [31] may prove to be an effective modality for teaching these skills. Such methods would enable this skill set to be practised in an *ex vivo* setting before transfer and practice in the authentic emergency department environment.

### Limitations

One of the limitations of this study was the use of physicians in the analysis team (one EP and one resident physician). We chose to do this as the interpretive description technique values presence of the immersive experience in the analysis, but we are aware that their clinical experiences may have affected their analysis. We attempted to mitigate the impact of this limitation via two methods including involving non-physicians in our research team and also performing a member check [32].

Another limitation of this study is that we only involved physicians from a single academic institution, and this may limit the transferability of our data to other contexts. Yet another limitation may have been our choice to only interview junior residents and attending physicians. The lack of senior residents who were in the liminal space between junior resident and attending may have affected the themes and teaching barriers noted in our study. That said, our academic institution involves physicians from two very disparate practice environments and we also included physicians who had a mixed academic/community practice, which may have lessened this limitation as well.

## Conclusion

Emergency department management is a critical skill for EPs, especially considering increasing acuity and patient volumes. Our study shows that educational strategies must consider the different perceptions of busyness, the need for staged progression, and the building of foundation skills to support more sophisticated skills. Finally, there is an identified need for a framework to help EPs teach this tacit ability, which may be grounds for further work in this area.
